# Performance of Sustainable Semi-Rigid Pavements: Optimizing High-Content Reclaimed Asphalt Pavement with Rejuvenators and Novel Grout Formulations

**DOI:** 10.3390/ma18214840

**Published:** 2025-10-23

**Authors:** Naeem Aziz Memon, Gulzar Hussain Jatoi, Giuseppe Loprencipe, Laura Moretti, Nur Izzi Md. Yusoff

**Affiliations:** 1 Faculty of Civil Engineering, Universiti Teknologi MARA (UiTM), Shah Alam 40450, Malaysia; naeemaziz@uitm.edu.my; 2 Department of Civil, Building and Environmental Engineering, Sapienza University of Rome, Via Eudossiana 18, 00184 Rome, Italy; gulzarhussain.jatoi@uniroma1.it (G.H.J.); laura.moretti@uniroma1.it (L.M.); 3 Department of Civil Engineering, Aror University of Art, Architecture, Design and Heritage, RCW Rohri Bypass, Sukkur 65170, Sindh, Pakistan; 4 Department of Civil Engineering, Universiti Kebangsaan Malaysia (UKM), Bangi 4360, Malaysia; izzi@ukm.edu.my

**Keywords:** reclaimed asphalt pavement (RAP), semi-rigid pavement, rejuvenator, cementitious grout, interaction effects, material synergy, pavement durability

## Abstract

Achieving sustainable pavement construction through high-content Reclaimed Asphalt Pavement (RAP) is a critical industry goal, but its implementation is frequently challenged by the reduced mechanical performance and durability inherent in such mixtures. This study evaluates the performance of semi-rigid pavements with RAP from 0% to 100%, a chemical rejuvenator, and four novel cementitious grout formulations (G1–G4). A comprehensive experimental program examined compressive strength, flexural strength, rutting resistance, fatigue life, and moisture sensitivity. Statistical analysis revealed that increasing RAP content significantly reduced all performance metrics. However, the primary innovation of this work lies in identifying strong interaction effects between key variables. The chemical rejuvenator effectively mitigated performance losses, with its benefits most pronounced at higher RAP contents (*p* ≤ 0.003). Among the Gi types, G3, containing a proprietary high-reactivity mineral additive, consistently achieved superior results; for instance, the R100-J-G3 regained over 70% strength of the virgin control mix (R0-NJ-G3). Notably, the interaction between RAP content and grout type (*p* ≤ 0.015) revealed that G3’s performance increased with RAP content, demonstrating its pivotal role in enabling technically viable 100% RAP mixtures. These findings underscore that the successful use of high-content RAP depends not just on individual components but on the optimized synergy between rejuvenator and grout selection, offering a validated pathway for technically viable pavements containing 100% RAP, reducing reliance on virgin materials and lowering environmental impact.

## 1. Introduction

The escalating global demand for transportation infrastructure has intensified pressures on natural resources and heightened environmental degradation [1,2]. Conventional road pavement construction heavily relies on virgin aggregates and petroleum-based binders in resource-intensive processes that generate significant waste [3]. Globally, road infrastructure consumes more than 50 billion tons of abiotic mineral resources annually, primarily aggregates, making it one of the largest extractive sectors by volume [1,3,4]. In addition, the production and use of asphalt binders account for the consumption of over 400 million tons of abiotic fossil resources each year, contributing substantially to greenhouse gas emissions and long-term depletion of non-renewable reserves [5,6,7]. In response, sustainable pavement technologies that maximize the use of recycled materials have gained prominence, as the road construction sector seeks to mitigate its environmental footprint. Several alternative approaches are being explored, such as incorporating construction and demolition waste, steel slag, and fly ash as supplementary aggregates and binders to reduce the extraction of virgin resources and minimize landfill disposal [5,6]. The adoption of warm-mix asphalt technologies has also proven effective, lowering production temperatures by 20–40 °C and thereby decreasing fuel consumption and greenhouse gas emissions during mixing and compaction [7,8,9,10]. In parallel, innovations such as bio-based rejuvenators and polymer-modified binders enhance mixture durability while reducing dependence on fossil-derived materials, aligning road engineering practices with circular economy principles [6,11,12]. Furthermore, the field is advancing toward multi-functional pavements by incorporating novel materials. For instance, Phase Change Materials (PCMs) are being actively investigated to regulate pavement temperature, which can mitigate urban heat island effects and reduce thermal cracking [13]. Concurrently, self-healing technologies, which use methods like encapsulated rejuvenators or induction heating, offer a pathway to autonomously repair microcracks, thereby extending pavement service life and reducing maintenance needs [14]. While these functional advancements are promising, a foundational challenge remains in ensuring the structural integrity and durability of pavement mixtures that incorporate high volumes of recycled materials like RAP, which is the primary focus of the present study. While these functional advancements are promising, a foundational challenge remains in ensuring the structural integrity and durability of pavement mixtures that incorporate high volumes of recycled materials like RAP, which is the primary focus of the present study. While these functional advancements are promising, a foundational challenge remains in ensuring the structural integrity and durability of pavement mixtures that incorporate high volumes of recycled materials like RAP, which is the primary focus of the present study. Within this spectrum of sustainable solutions, RAP has emerged as one of the most impactful strategies [5,14,15,16,17], offering a direct route to reduce landfill burden [18], conserve non-renewable resources, lower environmental impact [2], and lower construction costs [19].

RAP stands out as a critical and highly effective strategy for promoting sustainable infrastructure [20,21]. While RAP has been widely adopted in conventional flexible pavements, its integration into the distinct category of semi-rigid pavements, hybrid systems that combine the rutting resistance and bearing capacity of cement-stabilized bases with the flexibility of asphalt layers [4,22,23], remains constrained by the inherent stiffness and oxidation of the aged RAP binder, which reduces blend homogeneity and impairs interfacial cohesion with cementitious grouts [24,25,26]. Furthermore, the embrittlement and increased polarity of the aged binder exacerbate moisture damage, as the reduced adhesion between the binder and aggregate makes the mixture more susceptible to stripping [27]. These challenges often result in reduced interfacial bonding with cementitious grouts, thereby compromising mechanical strength and durability, particularly at high RAP contents. Indeed, laboratory studies report that the oxidized and high-performance-grade nature of RAP binder complicates both performance testing and the accurate determination of available binder content, thereby necessitating specialized rejuvenation or binder bump strategies to restore compatibility in semi-rigid systems [8,28,29,30]. These challenges often result in reduced interfacial bonding with cementitious grouts [31], thereby compromising mechanical strength and durability, particularly at high RAP contents [32]. Recent advancements have proposed two complementary solutions: the use of chemical rejuvenators to restore the viscoelastic properties of aged binder [8,28] and the development of innovative grout formulations with supplementary cementitious materials to enhance bonding and durability [24]. However, most prior studies have evaluated these strategies in isolation, leaving a critical knowledge gap regarding their interactive and synergistic effects when applied simultaneously in semi-rigid pavements with high or complete RAP substitution.

Addressing this gap, this study systematically examines the combined effect of a bio-oil-based rejuvenator and four innovative cementitious grout formulations on semi-rigid pavements with RAP levels ranging from 0% to 100%. By analyzing compressive and flexural strength, rutting resistance, fatigue life, and moisture sensitivity, this research measures the individual contributions of each component and explores their interdependent roles in restoring performance. This integrated approach fills a critical knowledge gap by investigating the complex and conditional relationships that influence the performance of this composite system, moving beyond a simple study to provide a comprehensive design framework. The findings deliver a detailed design approach for sustainable and environmentally friendly semi-rigid pavements, showing that with optimized rejuvenator–grout synergy, it is possible to build durable pavements using up to 100% RAP. This makes a significant contribution to sustainable infrastructure by reducing dependence on virgin materials, decreasing waste, lowering carbon emissions, and giving engineers and policymakers practical pathways toward greener, more cost-effective pavement solutions.

## 2. Materials and Methods

In this study, the semi-rigid pavement mixtures were formulated using crushed granite as fresh aggregates, RAP, a virgin asphalt binder, a bio-oil-based rejuvenator, and four cementitious grouts (G1, G2, G3, and G4). The asphalt skeleton was designed as an open-graded mixture to ensure the high porosity (between 25% and 35%) required for grout infiltration [24,33]. The blended gradation met Open-Graded Friction Course specifications ASTM D7064/D7064M [34] in Table 1.

The RAP material contained 3.7% by mass aged binder according to AASHTO T164 [35]. The recovered binder exhibited stiff, brittle behavior (e.g., low penetration and high viscosity) due to oxidation. Table 2 lists the physical properties of the RAP.

A softer virgin binder, Performance Grade (PG) 58-28, was selected to compensate for RAP stiffness, with penetration of 75 (0.1 mm) at 25 °C and viscosity of 0.45 Pa·s at 135 °C, compared to 21.5 and 0.83 Pa·s, respectively, for the recovered RAP binder (Table 3).

A commercially available, bio-oil-based rejuvenator (Sylvaros™: Kraton Corp., Houston, TX, USA) was used to restore aged binder properties based on its composition, rich in maltenes, which is effective in re-solubilizing asphaltene clusters, thereby restoring flexibility [8,28]. The rejuvenator was added at a dosage of 5% by weight of the aged RAP binder. This application rate was determined through preliminary trials as optimal to reduce viscosity without compromising rutting resistance.

Four grout formulations were designed by varying proportions of Ordinary Portland Cement (OPC) Type-I, Class F fly ash, and micro-silica to serve as the binding matrix composed of different supplementary cementitious materials. Micro-silica was included for its dual function as an ultrafine filler, which improves particle packing and reduces permeability, and as a highly reactive pozzolan, which consumes calcium hydroxide to form additional calcium-silicate-hydrate gel. G3 incorporated a proprietary blend to enhance bonding and durability through improved Interfacial Transition Zone (ITZ) performance, enhancing overall durability [31]. Table 4 lists the mix proportions for each grout.

It should be noted that the comparison between G2 and G3 involves changes in both micro-silica content and the presence of a special additive. Consequently, G3 is evaluated as a combined, high-performance system, and this study does not deconvolve the individual contributions of the increased micro-silica versus the proprietary blend.

A full-factorial experimental design was implemented with RAP contents of 0, 25, 50, 75, and 100% (i.e., R0, R25, R50, R75, and R100, respectively), grout types G1–G4, and rejuvenator conditions with and without addition (except R0 without rejuvenator), yielding 36 mix combinations (Table 5).

Based on the literature for similar open-graded mixtures, an Optimum Binder Content (OBC) of 4.0% by total weight of the open-graded asphalt skeleton of the control mix (R0) was selected [24,41]. For all subsequent mixes containing RAP, the total binder content was maintained at 4.0%. Therefore, the amount of added virgin binder was adjusted to account for the 3.7% binder within the RAP fraction. The approach assumes 100% activation and blending of the aged RAP binder with the virgin binder and does not treat the RAP as a “black rock”.

All asphalt mixtures were prepared at a mixing temperature of (e.g., 155 °C ± 5 °C) and compacted at a temperature of [e.g., 140 °C ± 5 °C]. These temperatures were kept constants for all mixtures to provide a consistent basis for comparison. The specimens were compacted using a Superpave Gyratory Compactor, with compactive effort adjusted according to RAP content to achieve a uniform air void content of 30 ± 1%. Due to the increased stiffness of the mixes [9,42,43], the number of design gyrations (N_design) increased with RAP content from 53 for R0 to 97 at R100 (Table 6).

For each of the 36 mix combinations, three replicate specimens were prepared and tested for each performance evaluation to ensure statistical validity. The mechanical and durability performances of the mixtures were assessed using five standard tests (Table 7). This suite of tests was selected to provide a comprehensive evaluation of the material’s structural capacity, resistance to primary distresses (rutting and fatigue), and environmental durability. While other tests such as the Indirect Tensile (IDT) strength test are valuable, the chosen methods were deemed sufficient to meet the study’s objectives within a manageable experimental scope.

A rigorous statistical analysis was performed using Statistical Package for the Social Sciences (SPSS) software v.28, employing regression models and multi-factor Analysis of Variance (ANOVA) to determine the significance of RAP content, grout type, and rejuvenator application, as well as their two-way interactions. Post hoc Tukey’s HSD tests were conducted where relevant to identify significant pairwise differences. Statistical significance was established at α = 0.05. The statistical population consists of all tested specimens.

## 3. Results

The performance of the semi-rigid pavement mixtures was evaluated across 36 mixtures to investigate the effects of the modified parameters on compressive strength, flexural strength, fatigue resistance, moisture resistance, and rutting susceptibility.

### 3.1. Experimental Results

**Figure 1:**
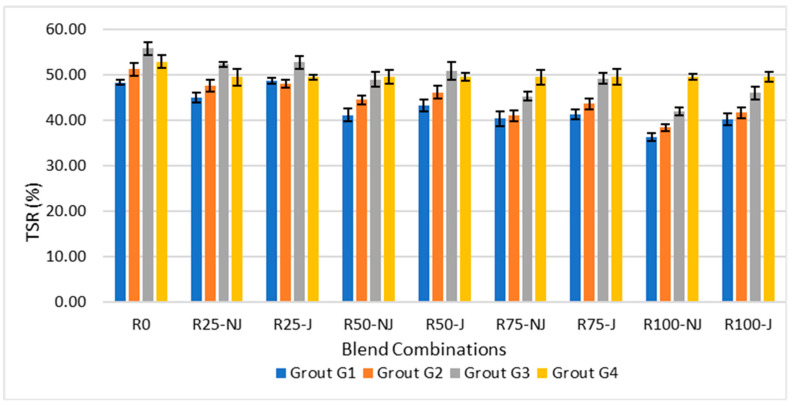
Compressive strength results for different RAP contents. (Note: NJ = Non-Rejuvenator, J = Rejuvenated).

Compressive strength (Figure 1) decreased markedly with higher RAP content, yet rejuvenated mixes consistently outperformed non-rejuvenated counterparts. Among grout formulations, G3 (grey bars) achieved the highest compressive strength values, particularly at high RAP contents, where it enabled substantial recovery of load-bearing capacity.

Increasing RAP content reduces the compressive strength by about 10–12 MPa (≈20–25%) across all grout types when going from 0% to 100% RAP. This decline is nearly parallel across all grout types, showing that the relative ranking is maintained regardless of RAP content. At all RAP content levels, Grout G1 (blue) has the minimum compressive strength. Whatever the RAP content, the maximum absolute difference between the strongest and weakest grout is around 10 MPa (e.g., at 0% RAP, ~55 MPa vs. ~48 MPa). The maximum percentage difference at 0% RAP is 14.6%; even at the highest RAP content (e.g., 100%), the difference remains significant, ranging between 7 and 9 MPa (i.e., 18–22%).

**Figure 2:**
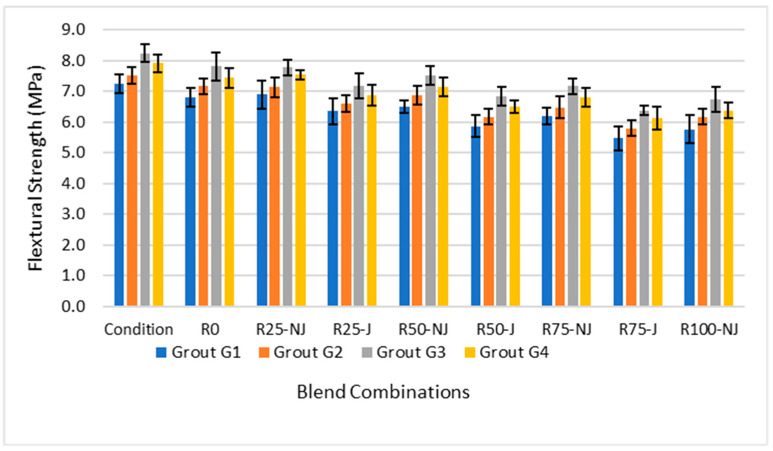
Flexural strength results for different RAP contents.

Flexural strength trends in Figure 2 confirmed the compressive results, with rejuvenation and G3 both contributing to significant improvements compared to other mix configurations.

Rejuvenated mixtures consistently outperformed non-rejuvenated mixtures, with average gains of about 8–12% across RAP contents. Among the grouts, G3 achieved the highest values, which represents a 6–9% improvement compared to G1. As confirmed by the multi-factor ANOVA (Table 8), the effects of both the rejuvenator and the grout type were found to be statistically significant, indicating these performance gains are not attributable to experimental error. At higher RAP levels (≥75%), NJ mixtures dropped below 6.0 MPa, while rejuvenated systems sustained strengths closer to 6.7–7.0 MPa, demonstrating that rejuvenation mitigates the negative impact of RAP incorporation.

**Figure 3:**
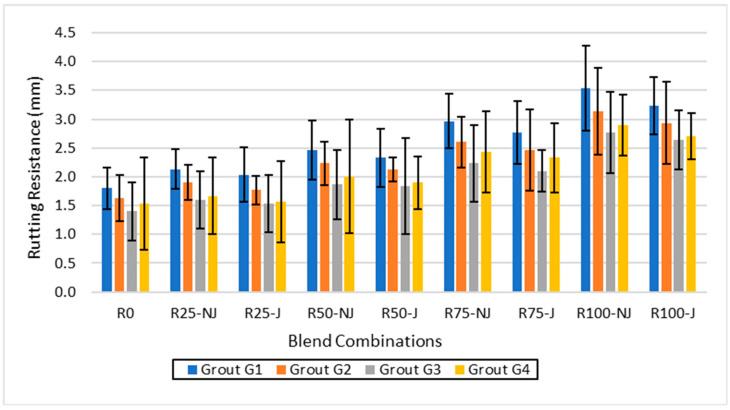
Rut depth results for different RAP contents.

Rutting performance (Figure 3) deteriorated with increased RAP content, but rejuvenated mixtures displayed smaller rut depths, reflecting enhanced deformation resistance.

Rut depths increased from approximately 1.6–1.8 mm at 0% RAP to more than 3.0 mm at 100% RAP in non-rejuvenated mixes. Rejuvenation consistently reduced rut depths by 10–15% compared to the corresponding non-rejuvenated mixtures. With 50% RAP, the rut depth decreased from about 2.5 mm (NJ) to 2.2 mm (J). Among the grouts, G3 exhibited the best rutting resistance, achieving the lowest rut depths across all RAP levels; at 100% RAP, G3 maintained the rut depth near 2.7 mm, which is around 12% lower than G1. G4 also performed well, though it was slightly lower than G3, while G1 consistently showed the highest rutting susceptibility. Overall, the combination of rejuvenation and G3 grout proved most effective in mitigating rutting damage, maintaining rut depths within acceptable limits even at high RAP contents.

**Figure 4:**
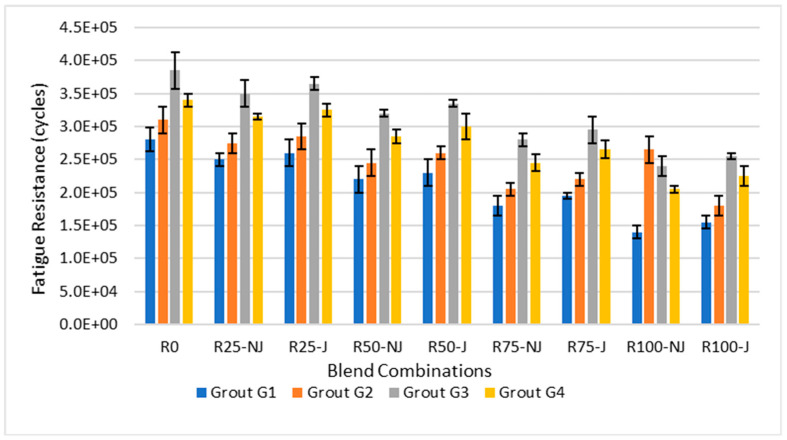
Cycles to failure for different RAP contents.

In Figure 4, the fatigue results reveal a sharp decline in cycles to failure with higher RAP, although the rejuvenator’s addition significantly extended fatigue life.

Figure 4 shows a clear reduction in fatigue resistance with increasing RAP. In non-rejuvenated mixtures, fatigue life decreased from about 380,000 cycles at 0% RAP to roughly 170,000 cycles at 100% RAP, representing a 55% reduction. Rejuvenation helped mitigate this loss, extending fatigue life by 20–30% across RAP levels. For example, at 50% RAP, the addition of rejuvenator increased resistance from approximately 220,000 cycles (NJ) to 290,000 cycles (J), a gain of nearly 32%.

Differences among the grouts were also significant. G3 consistently outperformed the others, achieving up to 25% higher fatigue life than G1 at equivalent RAP levels. Even at 100% RAP, G3 maintained around 230,000 cycles, compared with 170,000 cycles for G1 (+35%). G4 ranked second, while G2 showed moderate benefits. Overall, the data confirm that rejuvenation, coupled with G3, substantially improves flexibility and long-term durability, offsetting the stiffness-related fatigue losses caused by high RAP contents.

**Figure 5:**
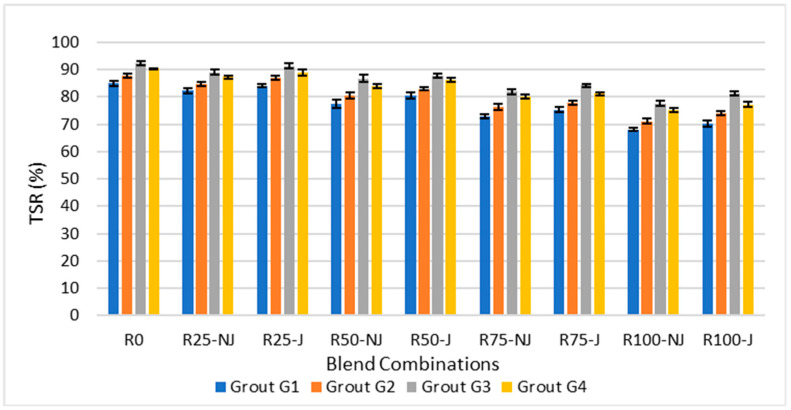
Moisture sensitivity for different RAP contents.

Figure 5 demonstrates that the Tensile Strength Ratio (TSR) decreased with increasing RAP content, reflecting reduced resistance to moisture damage.

In NJ mixtures, TSR dropped from around 88% at 0% RAP to nearly 65% at 100% RAP, a decline of approximately 26%. The addition of a rejuvenator improved moisture durability, raising TSR by 10–18% compared to NJ counterparts. When comparing at 50% RAP, rejuvenation increased TSR from about 73% (NJ) to 84% (J), corresponding to a 15% improvement.

Similarly, when analyzing grouts, G3 consistently achieved the highest TSR values, outperforming G1 by up to 12% at high RAP levels, which highlights its superior bonding and resistance to stripping. G4 followed closely behind, while G2 showed moderate improvements. Overall, the combination of rejuvenation and G3 grout provided the best defense against moisture-induced damage, maintaining TSR values above 80% even at elevated RAP contents.

### 3.2. Statistical Significance and Interaction Effects

The statistical analysis confirmed the observed trends from the experimental data. Table 8 presents the multi-factor ANOVA results, including the F-statistic, *p*-value, and partial eta-squared (η_p_^2^) for each source of variation.

**Figure 6:**
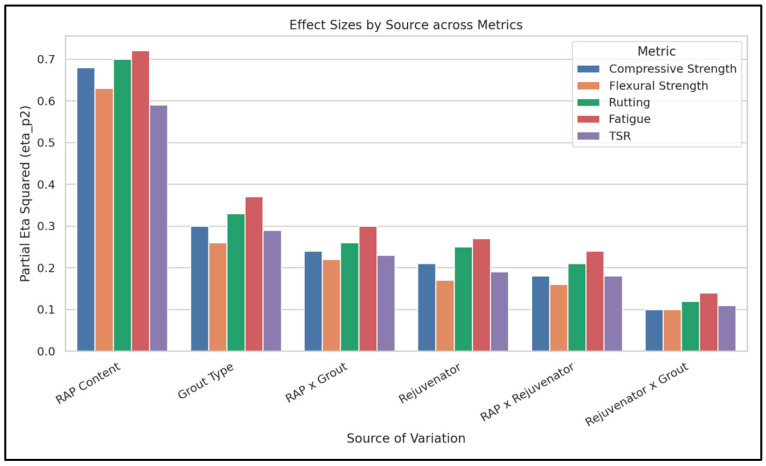
Summary of multi-factor ANOVA results for performance parameters.

The ANOVA results reveal that RAP content, grout type, and rejuvenator presence all exerted significant main effects (*p* < 0.001) on every measured parameter. The η_p_^2^ values indicated that RAP content was the most influential factor, explaining 59–72% of the variance. Figure 6 provides a summary of the significant influence of each factor, along with their interactions.

Importantly, significant two-way interactions were observed between RAP and grout type, RAP and rejuvenator, and rejuvenator and grout type (*p* ≤ 0.015), confirming that the effects of each factor were conditional on the others. To further explore the relationship between RAP content and performance, a regression and correlation analysis was conducted. Table 9 further reveals strong linear relationships between RAP content and performance metrics. The high correlation coefficients underscore the predictable impact of RAP content on material properties.

With Pearson correlation coefficients exceeding ∣0.82∣ in all cases, the analysis demonstrates a strong and predictable relationship between RAP content and the performance of the pavement mixtures. For instance, the negative correlation coefficients for compressive and flexural strength confirm that higher RAP content leads to a decrease in these properties. Finally, a post hoc analysis was performed to identify specific differences between the grout types, with the results summarized in Table 10.

Following the significant main effect of grout type, Tukey’s HSD post hoc comparisons were conducted to identify which specific grout types were statistically different from one another (Table 10).

The results from Table 10 demonstrate that grout type G3 provided significantly higher compressive strength than other grout types (*p* < 0.05), confirming its superiority as a binding matrix in RAP-rich semi-rigid pavements. Collectively, these statistical results highlight that although RAP incorporation reduces performance, the combined use of a chemical rejuvenator and an optimized grout, particularly G3, enables semi-rigid pavements with up to 100% RAP to achieve mechanical and durability properties comparable to control mixes with virgin materials.

## 4. Discussion

The results of this study provide a comprehensive analysis of the factors influencing the performance of semi-rigid pavements with high RAP content. The multi-factor ANOVA confirmed that RAP content, rejuvenator, and grout type all have a statistically significant main effect on performance metrics, a finding consistent with existing literature on sustainable pavement materials [4,8,24,25].

However, a more critical discussion of these findings reveals key quantitative insights. The η_p_^2^ values reveal that RAP content is the dominant factor, explaining a substantial 59% to 72% of the variance in the performance parameters. This effect size is notably larger than that of the rejuvenator (η_p_^2^ = 0.17–0.27) and grout type (η_p_^2^ = 0.26–0.37). This numerical dominance of RAP content underscores the primary challenge in using recycled materials: its inherent properties, particularly the aged binder, dictate a large portion of the final mix performance. Therefore, any successful mitigation strategy must effectively counteract this primary influence [23,24].

The existence of significant two-way interactions between all factors (e.g., RAP × rejuvenator, RAP × grout; *p* ≤ 0.015) is a crucial finding that moves beyond simple main effects. This supports the notion that a holistic mix design approach is essential. For instance, the optimal effect of a rejuvenator is not uniform across all RAP contents; it is conditional on the amount of aged binder present. Similarly, the effectiveness of a specific grout type depends on the RAP content, as confirmed by the study’s findings and supported by research on binder–aggregate interactions.

The regression analysis further quantifies the relationship between RAP content and performance. The strong correlation coefficients (e.g., r = −0.90 for fatigue) provide a high degree of confidence in the predictable decline of certain mechanical properties as RAP content increases. This is a common finding in pavement research, where increasing recycled material content is often associated with reduced fatigue life and moisture sensitivity due to the brittle nature of the aged binder. The correlation with rutting was negative (r = −0.88), and the performance worsened, which is contrary to typical findings in flexible. This suggests that a high RAP content decreases resistance to permanent deformation, likely due to the stiffer nature of the aged asphalt.

Finally, Tukey’s HSD post hoc analysis provides specific evidence for the superiority of grout type G3. Quantitatively, G3 yielded a compressive strength that was statistically higher than G1 by 4.55 MPa and G2 by 2.80 MPa (*p* < 0.05). This finding is critical as it validates the importance of selecting an optimized binding matrix to compensate for the reduction in performance from RAP.

The findings of this study both corroborate and extend the existing body of knowledge. These findings not only confirm established trends, such as the decline in mechanical performance with RAP and the efficacy of rejuvenators [33], but also extend the literature by providing systematic evidence of interactive synergies in semi-rigid pavements with up to 100% RAP, an area where data has been limited [8,24]. This study provides a more nuanced contribution by systematically evaluating the rheological properties and storage stability of semi-rigid pavements with up to 100% RAP content, an area where data is scarce [29]. The key novelty lies in the statistical identification and interpretation of the synergistic interactions between the rejuvenator, grout type, and RAP content. While previous work has often studied these components individually [28,49], this research demonstrates that their combined, interactive effect is the critical determinant of final performance. The findings provide engineers with a design framework based on material synergy: the choice of grout and the necessity of a rejuvenator are not independent decisions but should be optimized based on the intended RAP content. This approach facilitates the large-scale diversion of RAP from landfills, conserves virgin materials, and reduces the overall cost and environmental footprint of pavement construction [18].

It is crucial to contextualize the findings of this study within the specific scope of the materials investigated. The results and conclusions presented are directly applicable to semi-rigid pavements constructed with the specific open-graded gradation, single RAP stockpile, and conventional PG 58-28 virgin binder used herein. While the principles of material synergy between rejuvenators and advanced grouts are demonstrated, the quantitative outcomes may differ with other materials, such as different RAP sources with varying aged binder properties, different aggregate gradations (e.g., dense-graded), or the use of polymer-modified asphalt binders. Therefore, the direct generalization of these results should be approached with caution, and further research is warranted to validate these findings across a wider range of materials.

Nevertheless, limitations exist: laboratory conditions cannot fully replicate field environments, and the proprietary nature of G3 constrains mechanistic explanations. Future research should focus on full-scale field validation and advanced microstructural characterization of the ITZ. Such studies would deepen our understanding of synergistic mechanisms and accelerate the global adoption of sustainable pavement solutions [50]. Finally, a comprehensive life-cycle assessment and cost analysis would provide the quantitative data on economic and environmental benefits needed to drive widespread adoption by industry and regulatory agencies.

## 5. Conclusions

Based on the comprehensive experimental program and statistical analysis conducted in this study, the following key conclusions can be drawn:

- Increasing the RAP content was the most dominant factor affecting pavement performance, significantly reducing compressive strength, flexural strength, fatigue life, and moisture resistance.
- The application of the bio-oil-based rejuvenator proved highly effective in mitigating the detrimental effects of the aged RAP binder, with its benefits being most pronounced at higher RAP contents.
- Among the novel grout formulations, G3, which contained a proprietary high-reactivity mineral additive, consistently yielded the best performance across all tested metrics, including compressive strength, rutting resistance, and fatigue life.
- The central finding of this research is the critical synergistic effect between the rejuvenator and grout selection. The optimized combination of the rejuvenator and the G3 grout enabled the 100% RAP mixture to regain over 70% of the virgin control mix’s strength, providing a validated and technically viable pathway for constructing sustainable semi-rigid pavements with complete replacement of virgin materials.

## Figures and Tables

**Table 1:** Blended gradation for open-graded asphalt skeleton.

Sieve Size (mm)	Blended Gradation (% Passing)	Specification Limits (% Passing)
19.0	100	100
12.5	95	90–100
9.5	65	55–75
4.75	18	10–25
2.36	8	5–10
0.075	3	2–4

**Table 2:** Properties of fresh aggregate and RAP [35,36].

Property	Fresh Aggregate	RAP	Test Method
Specific Gravity (Bulk, g/cm^3^)	2.65	2.58	ASTM C127 [36]
Water Absorption (%)	0.8	1.5	ASTM C127 [36]
Aged Binder Content (%)	---	3.7	AASHTO T164 [35]

**Table 3:** Properties of virgin and recovered binder [37,38,39,40].

Property	Virgin Binder PG (58-28)	Recovered RAP Binder	Test Method
Penetration @ 25 °C (0.1 mm)	75	21.5	AASHTO T49 [37]
Viscosity @ 135 °C (Pa·s)	0.45	0.83	AASHTO T316 [38]
DSR (G^∗^/sinδ) at 64 °C (kPa)	2.1	4.8	AASHTO T 315 [39]
Softening Point (°C)	48.0	72.2	AASHTO T53 [40]

**Table 4:** Mix proportions of cementitious grout formulations.

Grout Type	Portland Cement (%)	Class F Fly Ash (%)	Micro-Silica (%)	w/c Ratio	Special Additives
G1	70	30	0	0.50	None
G2	65	30	5	0.45	None
G3	60	30	10	0.45	Proprietary Blend
G4	75	25	0	0.50	None

**Table 5:** Experimental design matrix overview.

Factor	Levels	Conditions Applied
RAP Content (%)	0, 25, 50, 75, 100	All levels
Rejuvenator	No (NJ), Yes (J)	NJ for R0; NJ and J for R25, R50, R75, and R100
Grout Type	G1, G2, G3, G4	All combinations

**Table 6:** Optimum binder content formulation and compactive effort.

Mix Category	Total Binder Content (%)	Added Virgin Binder (%)	N_Design Gyrations
R0	4.0	4.00	53
R25	4.0	3.08	59
R50	4.0	2.15	68
R75	4.0	1.23	73
R100	4.0	0.30	97

**Table 7:** Summary of test procedures [44,45,46,47,48].

Test	Test Standard	Specimen Type
Compressive Strength	ASTM C39 [44]	Cylindrical (100 mm × 200 mm)
Flexural Strength	ASTM C78 [45]	Beam (75 mm × 75 mm × 300 mm)
Rutting Resistance	AASHTO T324 [46]	Slab (300 mm × 300 mm × 50 mm)
Fatigue	AASHTO T321 [47]	Beam (75 mm × 75 mm × 300 mm)
Moisture Sensitivity	AASHTO T283 [48]	Cylindrical (100 mm × 200 mm)

**Table 8:** Summary of Multi-Factor ANOVA Results for Performance Parameters.

Source of Variation	DF	Compressive Strength (F, *p*, η_p_^2^)	Flexural Strength (F, *p*, η_p_^2^)	Rutting (F, *p*, η_p_^2^)	Fatigue (F, *p*, η_p_^2^)	TSR (F, *p*, η_p_^2^)
RAP Content	4	58.21, <0.001, 0.68	45.18, <0.001, 0.63	62.55, <0.001, 0.70	71.30, <0.001, 0.72	38.76, <0.001, 0.59
Rejuvenator	1	28.93, <0.001, 0.21	22.70, <0.001, 0.17	35.12, <0.001, 0.25	40.88, <0.001, 0.27	25.41, <0.001, 0.19
Grout Type	3	15.67, <0.001, 0.30	12.89, <0.001, 0.26	18.03, <0.001, 0.33	21.50, <0.001, 0.37	14.92, <0.001, 0.29
RAP × Rejuvenator	4	6.12, 0.001, 0.18	5.01, 0.003, 0.16	7.28, <0.001, 0.21	8.55, <0.001, 0.24	5.89, 0.001, 0.18
RAP × Grout	12	2.88, 0.008, 0.24	2.51, 0.015, 0.22	3.15, 0.005, 0.26	3.80, 0.001, 0.30	2.75, 0.010, 0.23
Rej. × Grout	3	4.50, 0.008, 0.10	3.95, 0.015, 0.10	5.10, 0.004, 0.12	5.90, 0.002, 0.14	4.25, 0.011, 0.11
Residuals	108	-	-	-	-	-

**Table 9:** Summary of regression and correlation analysis results (RAP Content vs. performance).

Performance Parameter	Regression Model (R^2^)	Pearson Correlation Coefficient (r)	*p*-Value
Compressive Strength	0.75	−0.87	<0.001
Flexural Strength	0.70	−0.84	<0.001
Rutting Resistance	0.78	−0.88	<0.001
Fatigue	0.82	−0.90	<0.001
Moisture Sensitivity	0.68	−0.82	<0.001

**Table 10:** Tukey’s HSD post hoc pairwise comparisons for grout type on compressive strength (MPa).

Comparison	Mean Difference	Std. Error	Adjusted *p*-Value	95% Confidence Interval
G3 vs. G1	4.55	0.89	<0.001	[2.79, 6.31]
G3 vs. G2	2.80	0.89	0.008	[1.04, 4.56]
G3 vs. G4	2.15	0.89	0.045	[0.39, 3.91]
G4 vs. G1	2.40	0.89	0.021	[0.64, 4.16]
G4 vs. G2	0.65	0.89	0.889	[−1.11, 2.41]
G2 vs. G1	1.75	0.89	0.152	[−0.01, 3.51]

## Data Availability

The original contributions presented in this study are included in the article. Further inquiries can be directed to the corresponding author.

## Abbreviations

The following abbreviations are used in this manuscript:

ITZ	Interfacial Transition Zone
TSR	Tensile Strength Ratio
RAP	Reclaimed Asphalt Pavement
ANOVA	Analysis of Variance
